# Toroidal Dipolar Excitation in Metamaterials Consisting of Metal nanodisks and a Dielectrc Spacer on Metal Substrate

**DOI:** 10.1038/s41598-017-00708-5

**Published:** 2017-04-03

**Authors:** Chaojun Tang, Bo Yan, Qiugu Wang, Jing Chen, Zhendong Yan, Fanxin Liu, Naibo Chen, Chenghua Sui

**Affiliations:** 1 Center for Optics & Optoelectronics Research and Department of Applied Physics, Zhejiang University of Technology, Hangzhou, 310023 China; 2 Department of Electrical and Computer Engineering, Iowa State University, Ames, Iowa 50011 USA; 3 College of Electronic Science and Engineering, Nanjing University of Posts and Telecommunications, Nanjing, 210023 China; 4 National Laboratory of Solid State Microstructures, Nanjing University, Nanjing, 210093 China

## Abstract

We have investigated numerically toroidal dipolar excitation at optical frequency in metamaterials whose unit cell consists of three identical Ag nanodisks and a SiO_2_ spacer on Ag substrate. The near-field plasmon hybridization between individual Ag nanodisks and substrate forms three magnetic dipolar resonances, at normal incidence of plane electromagnetic waves. The strong coupling among three magnetic dipolar resonances leads to the toroidal dipolar excitation, when space-inversion symmetry is broke along the polarization direction of incident light. The influences of some geometrical parameters on the resonance frequency and the excitation strength of toroidal dipolar mode are studied in detail. The radiated power from toroidal dipole is also compared with that from conventional electric and magnetic multipoles.

## Introduction

In 2010, dominant toroidal dipolar response was firstly experimentally observed at microwave, in metamaterials consisting of a three-dimensional (3D) array of four asymmetric split-ring resonators (SRRs)^1^. In 2012, toroidal dipolar response was then pushed theoretically to the optical frequency, by scaling down the size of SRRs^2^. The fabrication of 3D array of four asymmetric SRRs is not easy especially at optical wavelengths. In 2013, a simplified two-dimensional (2D) planar scheme was demonstrated in experiment for toroidal dipolar metamaterials which were also comprised of four asymmetric SRRs^3^. In the past several years, SRRs-based toroidal metamaterials have been drawing a lot of attentions^4–16^, thanks to their novel electromagnetic properties and a variety of potential applications such as low-threshold lasing^6^, polarization transformers^12^, electromagnetically induced transparency (EIT)^13^, and circular dichroism (CD)^16^.

Recently, toroidal dipolar response was also investigated in metamolecules with magnetic resonance^17–25^, plasmonic cavities^26–32^, and high-refractive-index dielectric nanostructures^33–41^. For example, the toroidal dipolar response in the optical regime was demonstrated experimentally in metamolecules that were formed by six pairs of asymmetric double-bars^17^. The toroidal dipolar response was also showed theoretically in metamolecules consisting of six gold disks on a gold substrate separated by a SiO_2_ layer, under the excitation of radially polarized light^22^. The theoretical and experimental evidence of toroidal dipolar response was presented in a plasmonic cavity comprising seven round holes drilled in a thick silver film^26^. A pronounced spectral feature in far-field scattering related to toroidal dipolar response was observed experimentally in high-refractive-index silicon nanoparticles, when the resonance frequencies of toroidal and electric dipole modes were tuned to be overlapped^39^.

In this work, we will theoretically study the excitation of toroidal dipolar mode at optical frequency in metamaterials composed of three Ag nanodisks with equal size and a SiO_2_ spacer on Ag substrate. It is found that under normal incidence of plane electromagnetic waves, the near-field plasmon hybridization between individual Ag nanodisks and substrate forms three magnetic dipolar resonances. The further strong coupling among three magnetic dipolar resonances will result into the excitation of toroidal dipolar mode, when space-inversion symmetry breaking is introduced in the polarization direction of incident light, through placing the Ag nanodisks in different locations. We have investigated in detail the influences of some geometrical parameters on the resonance frequency and the excitation strength of toroidal dipolar mode. The radiated power from toroidal dipole is also compared with that from conventional electric and magnetic multipoles. We hope that the numerical results presented in this work could be helpful to experimentally observe toroidal dipolar response at optical frequency.

## Results

**Figure 1:**
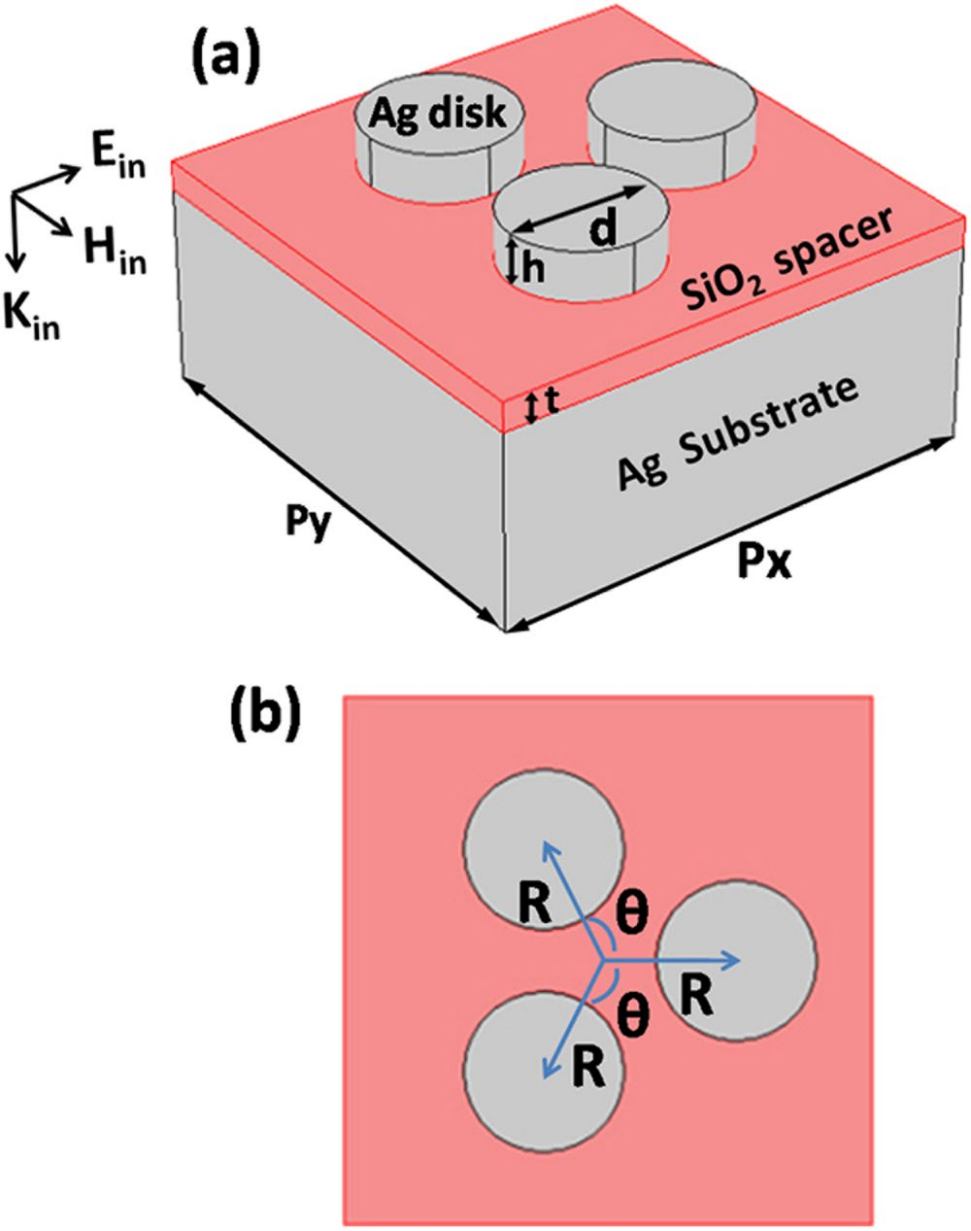
Oblique view (**a**) and top view (**b**) of metamaterials supporting a toroidal dipolar mode.

Figure 1 schematically shows the toroidal metamaterials composed of three Ag nanodisks and a SiO_2_ spacer on Ag substrate. *d* and *h* are the diameter and height of Ag nanodisks, and *t* is the thickness of SiO_2_ spacer. The relative positions of Ag nanodisks are determined by radius *R* and rotation angle *θ*. The periods along the *x* and *y* axes are *p*_*x*_ and *p*_*y*_. ***K***_***in***_, ***E***_***in***_, and ***H***_***in***_ are the wave vector, electric field, and magnetic field of incident light, respectively.

**Figure 2:**
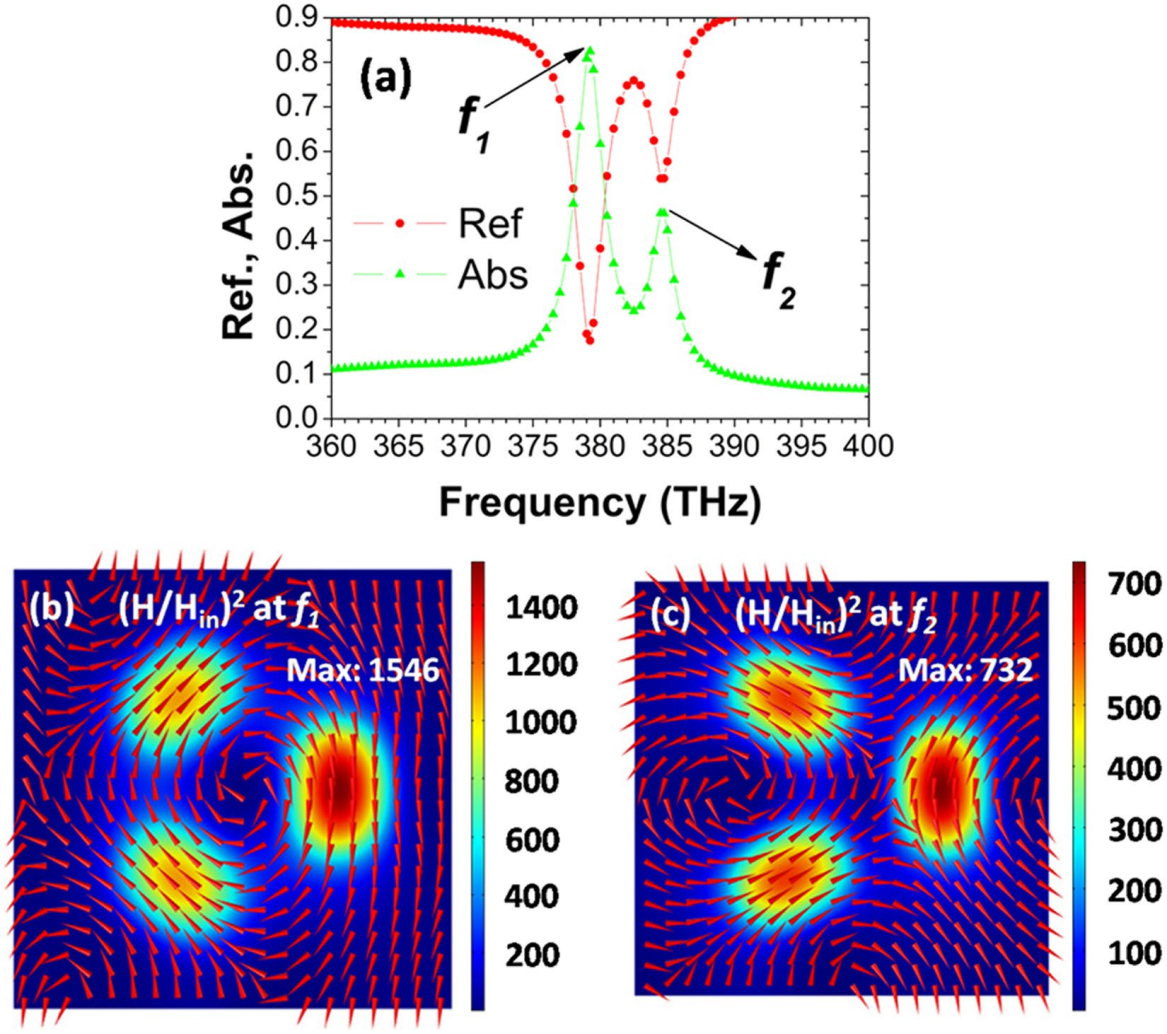
(**a**) Reflection and absorption spectra of toroidal metamaterials at normal incidence. (**b**,**c**) Magnetic field distributions on the *xy* plane across the center of SiO_2_ spacer, at the resonance frequencies of *f*_*1*_ and *f*_*2*_. Red arrows show the directions of magnetic fields, and colors give the intensity of magnetic fields. Geometrical parameters: *d* = 150 nm, *h* = 50 nm, *t* = 30 nm, *R* = 120 nm, *θ* = 120°, *p*_*x*_ = *p*_*y*_ = 500 nm.

Figure 2(a) shows the reflection (Ref., red circle) and absorption (Abs., green triangle) spectra of toroidal metamaterials under normal incidence of light, in the frequency range from 360 to 400 THz. The spectra are calculated by the commercial software package “EastFDTD”, which is based on finite-difference-time-domain (FDTD) method^42^. In our calculations, the relative permittivity of Ag is from experimental data^43^, and SiO_2_ has a refractive index of 1.45. In Fig. 2(a), there are two resonance modes centered at *f*_*1*_ = 379.25 THz and *f*_*2*_ = 384.75 THz, which correspond to wavelengths of *λ*_*1*_ = 791 nm and *λ*_*2*_ = 780 nm, respectively. At both *f*_*1*_ and *f*_*2*_ resonances, the reflection spectra have a dip, while the absorption spectra have a peak. To find the physical mechanisms of the resonant modes, Fig. 2(b–c) plot the magnetic field distributions at the resonance frequencies of *f*_*1*_ and *f*_*2*_. For resonant mode at *f*_*1*_, one can clearly see three field “hotspots” under Ag nanodisks. Moreover, the directions of magnetic fields have a head-to-tail distribution, which implies the excitation of a toroidal dipolar mode^1^. However, resonant mode at *f*_*2*_ does not have such a head-to-tail distribution, though there are also three field “hotspots”. It is well known that, the near-field plasmon hybridization between individual metal nanoparticle and a metal substrate can form a magnetic dipolar resonance^44, 45^, which has been widely explored for perfect absorption^46–48^. In our case, such plasmon hybridization forms three magnetic dipolar resonances under Ag nanodisks, resulting into the appearance of three field “hotspots”. In a similar approach reported in ref. 1, the further interactions among the magnetic dipolar resonances lead to the excitation of the toroidal dipolar mode.

**Figure 3:**
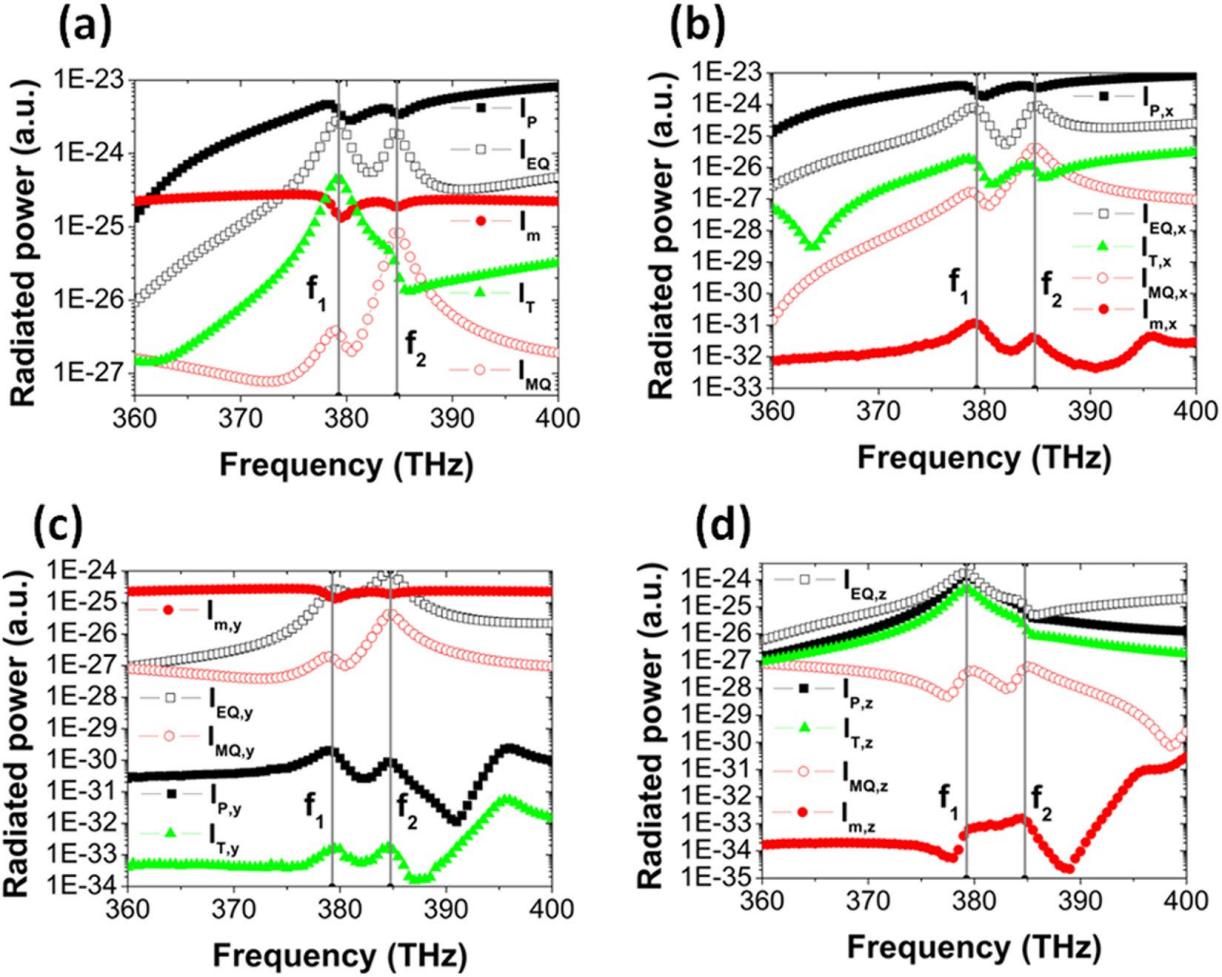
(**a**) Radiated power from electric dipolar moment (*I*_***p***_, solid black square), magnetic dipolar moment (*I*_***m***_, solid red circle), electric quadrupole dipolar moment (*I*_***EQ***_, hollow black square), magnetic quadrupole dipolar moment (*I*_***MQ***_, hollow red circle), and toroidal dipolar mement (*I*_***T***_, green triangle). (**b**,**c**) The same as (**a**) but for *x*, *y* and *z* components of radiated power, respectively. Two vertical gray lines label the resonance frequencies of *f*_*1*_ and *f*_*2*_ in Fig. 2(a).

To further demonstrate that resonant mode at *f*_*1*_ is closely related to the excitation of a toroidal dipolar mode, in Fig. 3 we have calculated the radiated power *I*_***p***_, *I*_***m***_, *I*_***EQ***_, *I*_***MQ***_, and *I*_***T***_ from electric dipolar moment ***p***, magnetic dipolar moment ***m***, electric quadrupole moment ***EQ***, magnetic quadrupole moment ***MQ***, and toroidal dipolar mement ***T***, respectively. In our calculations, the used equations^2^ are expressed as

1 $${\boldsymbol{p}}=(1/i\omega )\int \int \int {\boldsymbol{J}}\,{\rm{dv}}$$

2 $${\boldsymbol{m}}=(1/2c)\int \int \int ({\boldsymbol{r}}\times {\boldsymbol{J}})\,{\rm{dv}}$$

3 $${\boldsymbol{T}}=(1/10c)\int \int \int [({\boldsymbol{r}}.{J})r-{\bf{2}}{r}^{{\bf{2}}}{\boldsymbol{J}}]\,{\rm{dv}}$$

4 $${\boldsymbol{E}}{{\boldsymbol{Q}}}_{\alpha \beta }=(1/2i\omega )\int \int \int [({r}_{\alpha }{J}_{\beta }+{r}_{\beta }{J}_{\alpha })-2({\boldsymbol{r}}.{\boldsymbol{J}}){\delta }_{\alpha \beta }/3]\,{\rm{dv}}$$

5 $${\boldsymbol{M}}{{\boldsymbol{Q}}}_{\alpha \beta }=(1/3c)\int \int \int [{({\boldsymbol{r}}\times {\boldsymbol{J}})}_{\alpha }{r}_{\beta }+{({\boldsymbol{r}}\times {\boldsymbol{J}})}_{\beta }{r}_{\alpha }]\,{\rm{dv}}$$

6 $${I}_{{\boldsymbol{p}}}=(2{{\rm{\omega }}}^{4}/3{{\rm{c}}}^{3}){|{\boldsymbol{p}}|}^{2}$$

7 $${I}_{{\boldsymbol{m}}}=(2{{\rm{\omega }}}^{4}/3{{\rm{c}}}^{3}){|{\boldsymbol{m}}|}^{2}$$

8 $${I}_{{\boldsymbol{T}}}=(2{{\rm{\omega }}}^{6}/3{{\rm{c}}}^{5}){|{\boldsymbol{T}}|}^{2}$$

9 $${I}_{{\bf{EQ}}}=({{\rm{\omega }}}^{6}/5{{\rm{c}}}^{5})\sum {|{\boldsymbol{E}}{{\boldsymbol{Q}}}_{\alpha \beta }|}^{2}$$

10 $${I}_{{\bf{MQ}}}=({{\rm{\omega }}}^{6}/40{{\rm{c}}}^{5})\sum {|{\boldsymbol{M}}{{\boldsymbol{Q}}}_{\alpha \beta }|}^{2}$$

where ***r*** is position vector, ***J*** is volume current density, *ω* is frequency of incident light, *c* is light speed in vacuum, *i* is unit imaginary number, *dv* indicates the volume integration carried out in a unit cell, ∑ represents sigma summation, *δ*_*αβ*_ is delta function, and *α*, *β* = *x*, *y*, *z*. It is clearly seen in Fig. 3(a) that, the radiated power *I*_***T***_ from toroidal dipolar mement ***T*** has a peak exactly at *f*_*1*_, which clearly indicates that resonant mode at *f*_*1*_ is closely related with the excitation of a toroidal dipolar mode. Near the frequency of *f*_*1*_, the radiated power *I*_***T***_ is larger than the radiated power *I*_*m*_ from magnetic dipolar moment ***m*** and the radiated power *I*_***MQ***_ from magnetic quadrupole moment ***MQ***, but it is still smaller than the radiated power *I*_***p***_ from electric dipolar moment ***p*** and the radiated power *I*_***EQ***_ from electric quadrupole moment ***EQ***. By decomposing the *x*, *y* and *z* components of radiated power in Fig. 3(b–d), it is found that the *z* component *I*_***T***, ***z***_ dominates the radiated power *I*_***T***_, and it can be comparable with the *z* component *I*_***p***,***z***_ and *I*_***EQ***,***z***_, as shown in Fig. 3(d).

## Discussion

**Figure 4:**
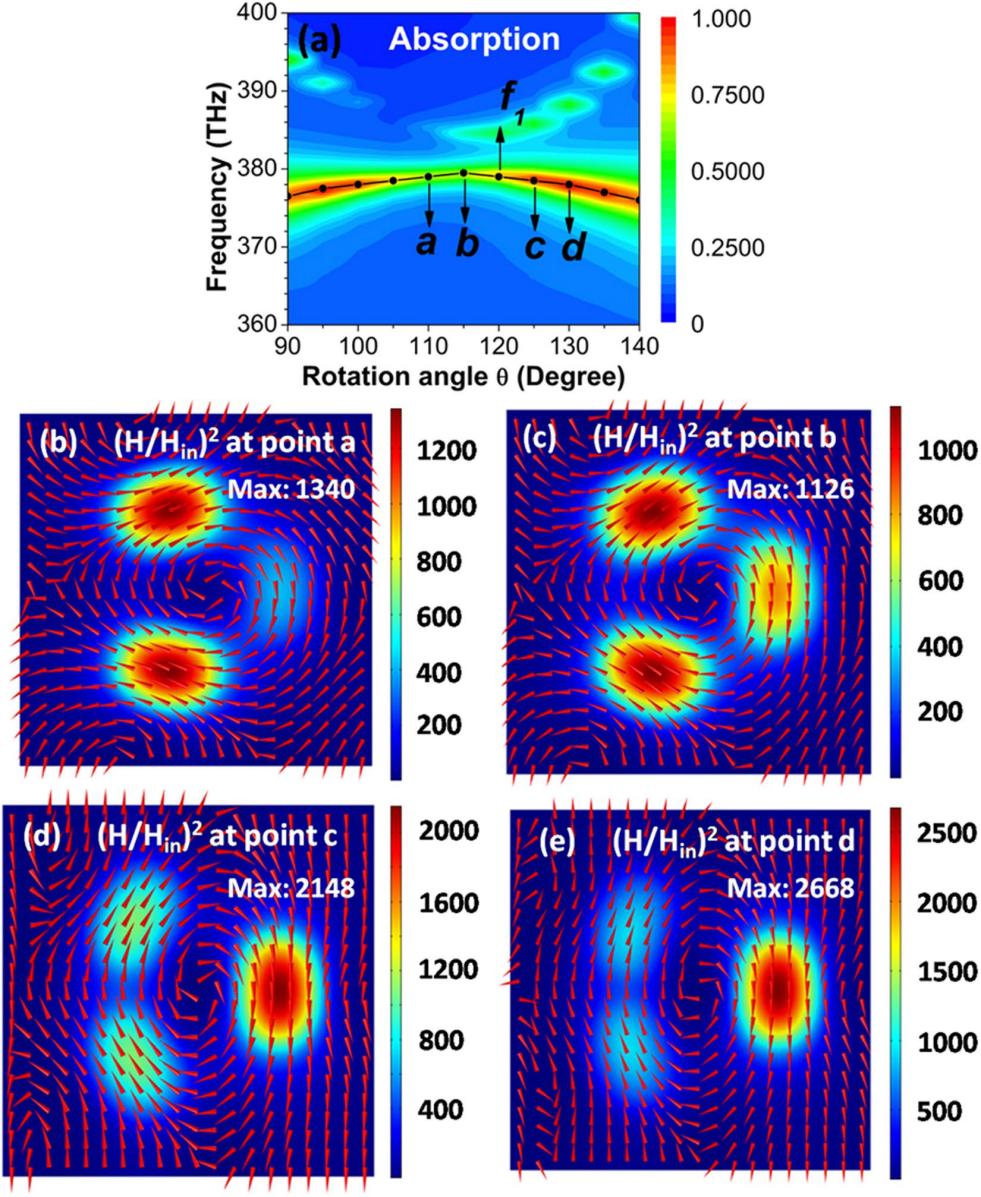
(**a**) Contour plot of absorption spectra of toroidal metamaterials as a function of light frequency and rotation angle *θ* at normal incidence. The overlaid black line and solid circles give the resonance position of toroidal dipolar mode. (**b**–**e**) Magnetic field distributions on the *xy* plane across the center of SiO_2_ spacer, at *a*, *b*, c, and *d* points. Red arrows show the directions of magnetic fields, and colors give the intensity of magnetic fields.

To study the influence of rotation angle *θ* on the toroidal dipolar mode, we present in Fig. 4(a) the contour plot of absorption spectra of toroidal metamaterials as a function of light frequency and rotation angle *θ*. The toroidal dipolar mode will blue-shift until *θ* increases to about 115°, because of the continuously strengthened interactions of magnetic dipolar resonances between the left two nanodisks and the right one. But, it will have a red-shift when *θ* is further increased, since the left two nanodisks’ interactions are gradually weakened with increasing *θ*. Figure 4(b–e) show the magnetic field distributions on the *xy* plane across the center of SiO_2_ spacer at *a*, *b*, c, and *d* points, respectively. For these points, the directions of magnetic fields under Ag nanodisks also have a vortex distribution (i.e., a head-to-tail distribution), a character of toroidal dipolar mode. As exhibited in Fig. 4(c), three field “hotspots” are simultaneously the most obvious, suggesting a relatively stronger excitation of toroidal dipolar mode for *θ* to be about 115°. The right field “hotspot” in Fig. 4(a) and the left two field “hotspots” in Fig. 4(d) become much weaker, which indicates a weak excitation of toroidal dipolar mode. When *θ* is smaller than 110° or larger than 140°, in principle, it is not a toroidal resonance and just is a magnetic dipole resonance.

**Figure 5:**
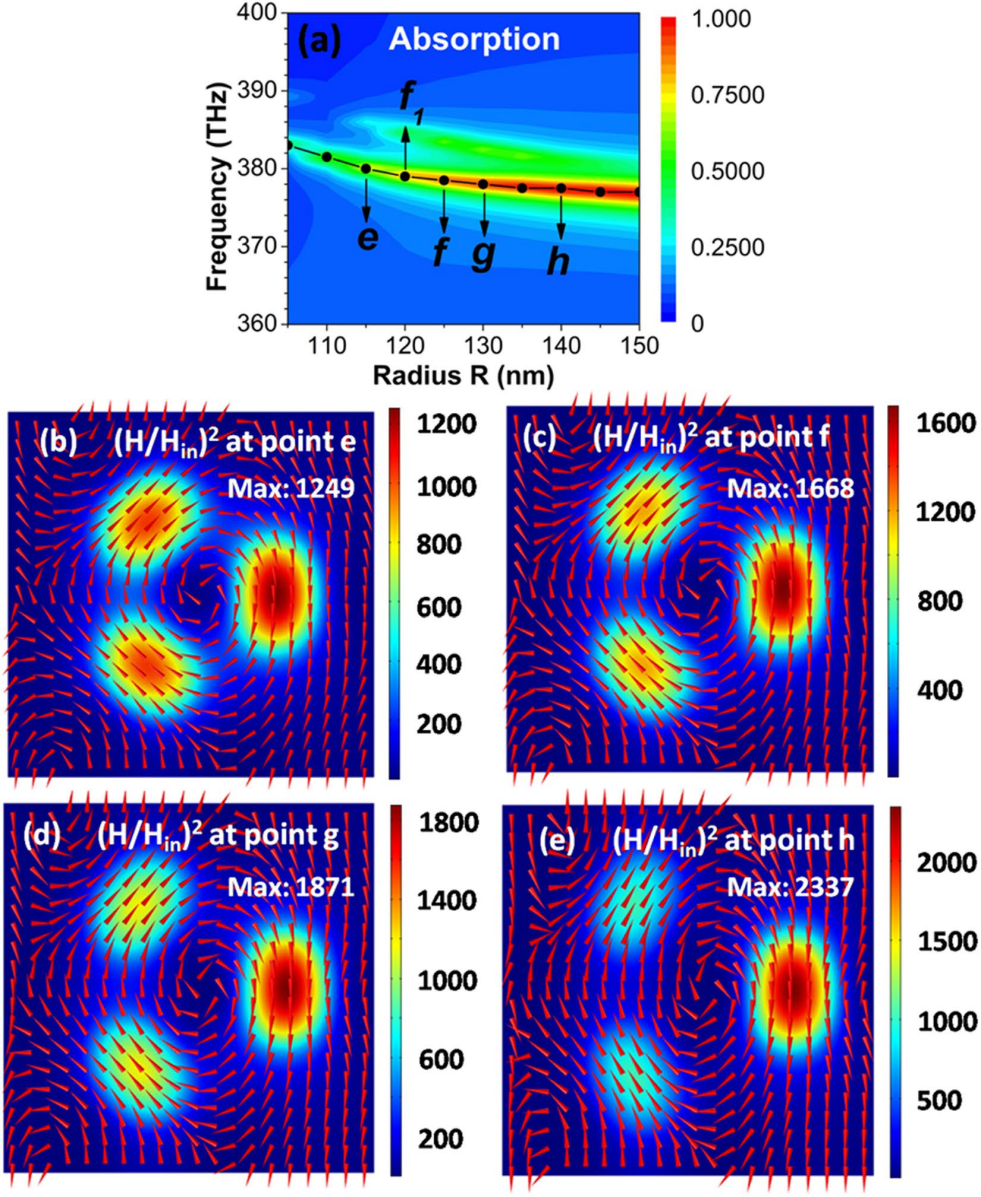
(**a**) Contour plot of absorption spectra of toroidal metamaterials as a function of light frequency and radius *R* at normal incidence. The overlaid black line and solid circles give the resonance position of toroidal dipolar mode. (**b**–**e**) Magnetic field distributions on the *xy* plane across the center of SiO_2_ spacer, at *e*, *f*, g, and *h* points. Red arrows show the directions of magnetic fields, and colors give the intensity of magnetic fields.

We have also investigated the influence of radius *R* on the toroidal dipolar mode. Figure 5(a) shows the contour plot of absorption spectra of toroidal metamaterials as a function of light frequency and radius *R*. The toroidal dipolar mode is obviously red-shifted as *R* is varied from 105 to 150 nm, since the interactions of magnetic dipolar resonances among Ag nanodisks become weak with increasing *R*. Figure 5(b–e) show the magnetic field distributions on the *xy* plane across the center of SiO_2_ spacer at *e*, *f*, g, and *h* points, respectively. At the four points, the directions of the magnetic fields under Ag nanodisks all have a head-to-tail distribution, indicating the excitation of a toroidal dipolar mode. When radius *R* is increased further, the right field “hotspot” will get stronger, while the left two field “hotspots” will get weaker.

In conclusion, we have theoretically studied the excitation of toroidal dipolar mode at optical frequency in metamaterials composed of three Ag nanodisks and a SiO_2_ spacer on Ag substrate. The Ag nanodisks have identical size, but are placed in different locations to break space-inversion symmetry in the polarization direction of incident light. Under normal incidence of linearly polarized light, the near-field plasmon hybridization between individual Ag nanodisks and substrate forms three magnetic dipolar resonances, and their further interactions lead to the excitation of toroidal dipolar mode. We have investigated in detail the influences of some geometrical parameters on the resonance frequency and the excitation strength of toroidal dipolar mode. The radiated power from toroidal dipole is also compared with that from conventional electric and magnetic multipoles. Our designed metamaterials may be helpful to experimentally observe toroidal dipolar response at optical frequency.
